# Cadherin 2/4 signaling via PTP1B and catenins is crucial for nucleokinesis during radial neuronal migration in the neocortex

**DOI:** 10.1242/dev.132456

**Published:** 2016-06-15

**Authors:** Isabel Martinez-Garay, Cristina Gil-Sanz, Santos J. Franco, Ana Espinosa, Zoltán Molnár, Ulrich Mueller

**Affiliations:** 1 Molecular and Cellular Neuroscience Department, Dorris Neuroscience Center, The Scripps Research Institute, La Jolla, CA 92037, USA; 2 Department of Pediatrics, University of Colorado School of Medicine, Aurora, CO 80045, USA; 3 Program of Pediatric Stem Cell Biology, Children's Hospital Colorado, Aurora, CO 80045, USA; 4 Department of Physiology, Anatomy and Genetics, University of Oxford, Oxford OX1 3QX, UK

**Keywords:** CDH2, LIS1, Cadherin, Catenin, Nucleokinesis, Radial migration, Mouse

## Abstract

Cadherins are crucial for the radial migration of excitatory projection neurons into the developing neocortical wall. However, the specific cadherins and the signaling pathways that regulate radial migration are not well understood. Here, we show that cadherin 2 (CDH2) and CDH4 cooperate to regulate radial migration in mouse brain via the protein tyrosine phosphatase 1B (PTP1B) and α- and β-catenins. Surprisingly, perturbation of cadherin-mediated signaling does not affect the formation and extension of leading processes of migrating neocortical neurons. Instead, movement of the cell body and nucleus (nucleokinesis) is disrupted. This defect is partially rescued by overexpression of LIS1, a microtubule-associated protein that has previously been shown to regulate nucleokinesis. Taken together, our findings indicate that cadherin-mediated signaling to the cytoskeleton is crucial for nucleokinesis of neocortical projection neurons during their radial migration.

## Abstract (precis)

**Highlighted article:** In radially migrating mouse cortical neurons, cadherin-mediated signaling to the cytoskeleton regulates the forward movement of the nucleus.

## INTRODUCTION

The mammalian neocortex processes sensory information, controls motor output, and mediates higher cognitive functions. Excitatory neurons of the neocortex are largely generated in the ventricular zone (VZ) and subventricular zones (SVZ) of the dorsal pallium. From their place of birth, these neurons migrate radially to establish neocortical cell layers. Neurons that are destined for deep layers are born and migrate first and are then surpassed by later-born upper layer neurons (Haubensak et al., 2004; Noctor et al., 2001, 2004; Rakic, 1975). Deep and upper layer neurons use distinct forms of motility to reach their final position. Neurons that will populate deep layers V and VI migrate at a time when the cortical plate (CP) is still relatively thin. These neurons extend their leading processes into the marginal zone (MZ) and translocate their soma along these processes in a migration mode called somal translocation (Nadarajah et al., 2001). Upper layer neurons destined for layers II-IV migrate later in development when the CP has reached considerable thickness by the addition of layer V and VI neurons. As a consequence, the leading processes of later-migrating neurons cannot reach the MZ and they instead progress through three sequential stages of motility: multipolar migration in the intermediate zone (IZ) followed by glia-guided motility within the CP, and finally glia-independent somal translocation nearer the pial surface (Nadarajah et al., 2001; Noctor et al., 2004; Tabata and Nakajima, 2003; Tabata et al., 2009). Although substantial progress has been made in defining the mechanisms that regulate radial migration, we still know little about the neuronal cell surface receptors that are involved in different forms of motility. The mechanisms by which these cell surface receptors regulate the signaling pathways crucial for leading process extension and the subsequent translocation of the cell body and the nucleus (nucleokinesis) are also largely not understood.

An important feature of cell motility is the formation of adhesion contacts between the leading processes of migrating cells and their environment. These dynamic contacts provide focal points for transient cytoskeletal assembly and traction points that are crucial for force generation. Throughout their migration, the leading processes of projection neurons establish connections with different cell types, including radial glial cells (RGCs) during glial-guided motility and Cajal–Retzius cells during somal translocation (Edmondson and Hatten, 1987; Gil-Sanz et al., 2013; Gregory et al., 1988; Nadarajah et al., 2001; Noctor et al., 2004; Rakic, 1972; Tabata and Nakajima, 2003). Migrating neurons use adhesion receptors of the cadherin and Ig superfamilies to attach their leading processes to Cajal–Retzius cells during somal translocation (Gil-Sanz et al., 2013). The formation of these adhesion complexes is regulated by the reelin signaling pathway and involves the cytoplasmic adaptor protein afadin (also known as MLLT4) and the GTPase RAP1 (Franco et al., 2011; Gil-Sanz et al., 2013). Intriguingly, CDH2, but not reelin, is also implicated in glial-guided motility (Kawauchi et al., 2010). However, the upstream regulators and downstream signaling pathways by which cadherins regulate glial-guided motility are unclear.

Here, we demonstrate that CDH2 and CDH4 cooperate to regulate radial migration in the neocortex through the engagement of catenin proteins in a process that is regulated by PTP1B (also known as PTPN1). Strikingly, cadherin signaling is not essential for the extension of the leading process in migrating neurons. Instead, CDH2/4-mediated signaling is crucial for maintaining stable adhesion and cytoskeletal organization in migrating neurons, which are essential for centrosome movement and nucleokinesis.

## RESULTS

### Cadherin expression in the developing neocortex

**Fig. 1.**
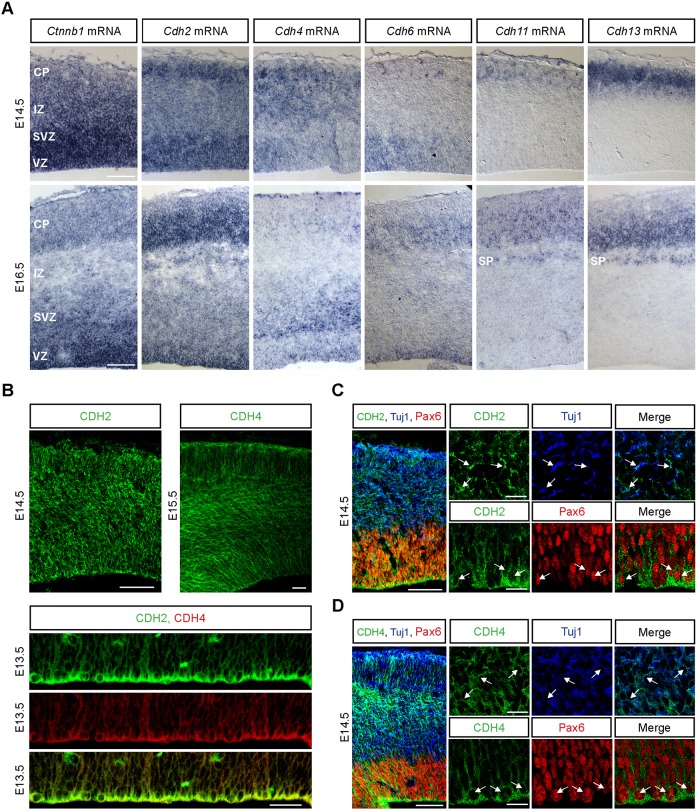
**Expression patterns of classical cadherins in the developing mouse lateral neocortex.** (A) *In situ* hybridization for β-catenin, *Cdh2*, *Cdh4*, *Cdh6*, *Cdh11* and *Cdh13* was carried out on coronal sections of E14.5 and E16.5 brains. Examples are shown from the developing somatosensory cortex. (B-D) Immunohistochemistry for CDH2 (green) and CDH4 (green; red in B, bottom panels) on coronal sections of the developing neocortex. The sections were co-stained with PAX6 (red) and TUJ1 (blue) antibodies (C,D). (B) CDH2 and CDH4 are expressed along the width of the cortical primordium. Both proteins are expressed in the ventricular zone, although CDH4 at lower levels than CDH2. (C) Both PAX6^+^ RGCs and TUJ1^+^ neurons express CDH2. (D) CDH4 is expressed by PAX6^+^ and TUJ1^+^ cells. Small panels in C and D are taken from different images that come from the same experiments. Arrows in C and D point to cells co-expressing the two markers. SP, subplate. Scale bars: 10 μm (C,D, small panels); 50 μm (all other panels).

Previous studies using a dominant-negative cadherin (DN-CDH) that interferes with the function of nearly all classical cadherins have revealed a role for cadherins in the radial migration of neocortical projection neurons (Franco et al., 2011; Gil-Sanz et al., 2013; Jossin and Cooper, 2011; Kawauchi et al., 2010). RNA interference experiments suggest that CDH2 is one of the classical cadherins important for radial migration (Franco et al., 2011; Gil-Sanz et al., 2013; Jossin and Cooper, 2011; Kawauchi et al., 2010). We wondered whether CDH2 acts in concert with other classical cadherins to regulate radial migration in mouse. *In situ* hybridization with probes for nearly all classical cadherins revealed that *Cdh2*, *Cdh4* and possibly *Cdh6* showed expression patterns consistent with a role in migration (Fig. 1A; data not shown). At embryonic day (E) 14.5, *Cdh2* as well as β-catenin (*Ctnnb1*) mRNA were detected along the width of the cortical wall (Fig. 1A). *Cdh4* mRNA, although detectable in the VZ, was more prominent in the IZ and CP, consistent with a role in migration. *Cdh6* mRNA was only visible in the VZ and SVZ, with a few isolated cells expressing *Cdh6* in the CP (Fig. 1A). *Cdh6* thus could perhaps act at the onset of migration, but probably not during later stages of migration when neurons have invaded the CP. Expression of other cadherins, such as *Cdh11* and *Cdh13*, was observed in the CP and SP (Fig. 1A), consistent with onset of expression in neurons during very late stages of migration or after migration is finished. At E16.5, expression of *Ctnnb1* and *Cdh2* was still strong throughout the cortical wall (Fig. 1A). *Cdh4* mRNA could be detected at lower levels than *Cdh2* with the strongest expression in the SVZ and IZ (Fig. 1A). *Cdh6* was expressed in the VZ and the CP, but only at very low levels in the SVZ and IZ (Fig. 1A). *Cdh11* and *Cdh13* strongly labeled cells in the SP and the CP (Fig. 1A).

Immunostainings at E14.5 confirmed that CDH2 and CDH4 were expressed throughout the cortical wall (Fig. 1B, upper panels). CDH2 was expressed in PAX6^+^ RGCs (Fig. 1C) as well as in TUJ1^+^ (TUBB3) neurons (Fig. 1C). CDH4 was also present in migrating neurons and RGCs, although at comparatively lower levels, especially at the ventricular surface where adherens junctions are present (Fig. 1B bottom panels; Fig. 1D). Specificity of the CDH2 and CDH4 antibodies was assessed both in brain sections from knockout animals and in heterologous cells transfected with HA-tagged cadherins (Figs S1, S2). These findings suggest that the two cadherins are good candidates to mediate the cell-cell interactions that are crucial for the radial migration of neocortical projection neurons.

### Both CDH2 and CDH4 contribute to radial migration

**Fig. 2.**
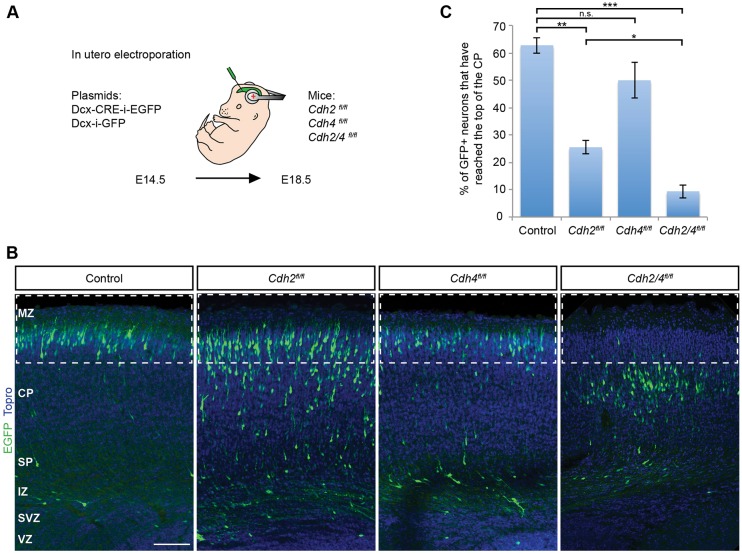
**CDH2 and CDH4 are required for radial migration in mouse cortex.** (A) Illustration of the strategy to inactivate *Cdh2* and *Cdh4* in migrating neurons. Embryos from floxed animals were electroporated *in utero* at E14.5 with DCX-Cre-i-EGFP or DCX-i-GFP. Position of the electroporated cells was analyzed at E18.5 in the developing somatosensory cortex. (B) Representative images of coronal sections of embryos electroporated as described in A. Electroporated neurons are shown in green and nuclei in blue (TO-PRO). (C) Quantification of the percentage of electroporated neurons that enter the boxed areas in B, representing the upper 25% of the CP. Four animals (*Cdh2^fl/fl^*), five animals (*Cdh4^fl/fl^*) and six animals (*Cdh2/4^fl/fl^*) from three separate experiments were analyzed for each condition. The data represent mean±s.e.m. n.s., non significant, *P<0.01, **P<0.0001, ***P<1×10^−13^ by Bonferroni post-hoc analysis after one-way ANOVA. MZ, marginal zone; SP, subplate. Scale bar: 100 μm.

To determine the extent to which CDH2 and CDH4 regulate radial migration, we obtained mice carrying a floxed allele of *Cdh2* (Kostetskii et al., 2005). We also generated mice carrying a floxed allele of *Cdh4* (Fig. S3)*.* We used *in utero* electroporation to introduce a plasmid expressing Cre recombinase and EGFP into embryos carrying floxed alleles for *Cdh2* and *Cdh4* (Fig. 2A). Expression of Cre and EGFP were controlled by a doublecortin (DCX) promoter, which is active in migrating neurons but not in RGCs (Franco et al., 2011; Wang et al., 2007). This allowed us to address the cell-autonomous functions of *Cdh2* and *Cdh4* in migrating neurons and to prevent disruption of adherens junctions between RGCs that are formed by CDH2 (Kadowaki et al., 2007).

Embryos were electroporated at E14.5 with DCX-Cre and the position of the electroporated neurons was determined at E18.5 using EGFP fluorescence. Four days after electroporation with DCX-Cre, CDH2 and CDH4 levels were reduced by about 36% and 47%, respectively (Fig. S4), indicating that gene function was partially but not completely compromised. By E18.5, more than half of the control cells, which expressed only EGFP, had migrated to a position near the cortical MZ (Fig. 2B,C). A higher number of neurons with reduced levels of either *Cdh2* or *Cdh4* remained in the IZ, SP and lower part of the CP, indicative of defects in radial migration (Fig. 2B,C). Strikingly, when we inactivated *Cdh2* and *Cdh4* simultaneously, only ∼10% of the electroporated cells reached their normal position near the cortical MZ. Instead, the cells accumulated within the inner portion of the CP (Fig. 2B,C). We conclude that both *Cdh2* and *Cdh4* contribute to the regulation of radial migration.

### Cadherin adhesive function is crucial for radial migration

**Fig. 3.**
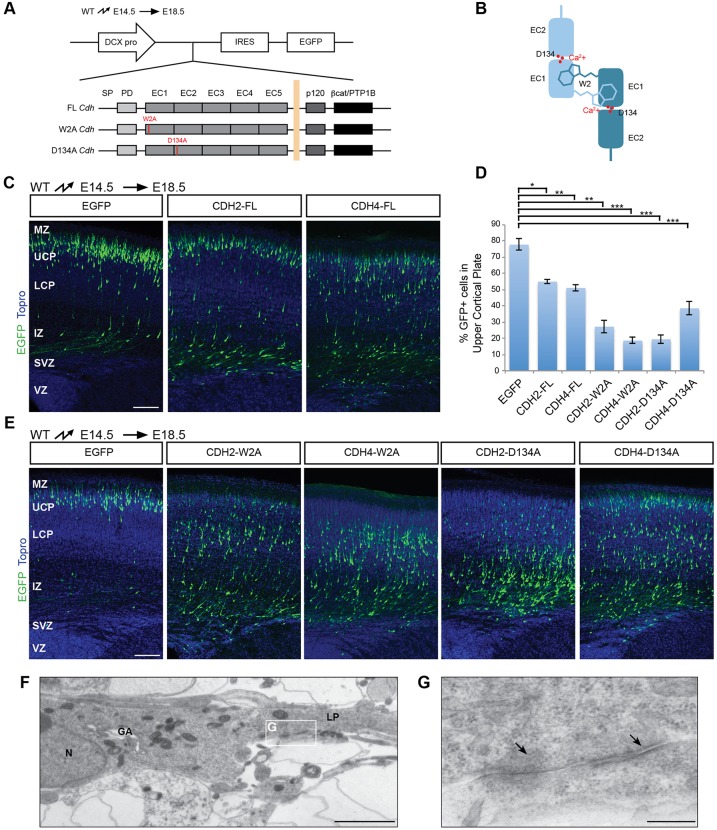
**Perturbation of CDH2/4 adhesive strength.** (A) Full length (FL) cDNAs encoding wild-type CDH2 and CDH4 or mutant proteins (W2A, D134A) were expressed in migrating neurons at E14.5 using *in utero* electroporation. The position of electroporated cells was determined at E18.5. DCX-pro, *Dcx* promoter fragment. (B) Diagram of the interaction between two cadherin molecules in trans. The first two cadherin repeats are shown for each molecule, and the critical Trp residue in position 2 is depicted inserting into the first EC domain of the opposing cadherin. Ca^2+^ ions are shown as red dots in between the extracellular (EC) domains and the approximate position of Asp134 is indicated. (C) Overexpression of full-length CDH2 or CDH4 impairs migration. Electroporated neurons are shown in green, TO-PRO-stained nuclei in blue. (D) Quantification (mean±s.e.m.) of the percentage of neurons reaching the upper half of the CP in C and E. *P<0.001, **P<0.0001, ***P<1×10^−7^ by Bonferroni post-hoc analysis after one-way ANOVA. For each condition, neurons were counted in three brain slices from each of four animals obtained from three independent electroporations. (E) Overexpression of adhesion-deficient cadherins (W2A, D134A) affects migration. Electroporated neurons are shown in green, TO-PRO-stained nuclei in blue. (F) Electron micrograph of a radially migrating neuron in the lower CP of an E16.5 cortex. The Golgi apparatus (GA) is localized in front of the nucleus (N), at the base of the leading process (LP). Adherens junction-like structures are visible between the leading neuronal process and a thin RGC process. (G) Higher magnification of the boxed area in F. Adherens junction-like structures appeared as darkened patches of membrane surrounded by a cloud of electron-dense material (arrows). LCP, lower cortical plate; MZ, marginal zone; UCP, upper cortical plate. Scale bars: 100 μm (C,E); 2 μm (F); 250 nm (G).

Previous studies suggest that regulation of cadherin levels at the cell surface is crucial for radial neuronal migration (Kawauchi et al., 2010), possibly by precise regulation of adhesive strength. Consistent with this model, we observed that overexpression of wild-type CDH2 or CDH4 in migrating neurons affected their migration; fewer cells reached the MZ by E18.5 (Fig. 3C,D), an effect that has been attributed to enhanced adhesive strength (Kawauchi et al., 2010; Shikanai et al., 2011). To test more rigorously whether cadherin adhesive function is required for radial migration, we generated expression vectors for CDH2 and CDH4 with point mutations in their extracellular domains (Fig. 3A) that disrupt cadherin adhesive function in cell aggregation assays (Ozawa et al., 1990), although low residual adhesive activity can be measured for both mutations in bead-aggregation assays using purified recombinant cadherins (Prakasam et al., 2006). One of the mutations, W2A, converts a critical Trp residue within the cadherin-cadherin interaction domain to an Ala (Shapiro et al., 1995; Tamura et al., 1998). The second mutation, D134A, lies outside the cadherin-cadherin binding domain but is important for calcium binding (Alattia et al., 1997; Pokutta et al., 1994) (Fig. 3B). We expressed full-length CDH2 or CDH4 constructs carrying the mutations by *in utero* electroporation in E14.5 brains under control of the DCX promoter and analyzed cell position at E18.5. Similar to the results obtained when expressing Cre in neurons carrying *Cdh2/4* floxed alleles (Fig. 2B,C), expression of all four mutant proteins (CDH2-W2A, CDH4-W2A, CDH2-D134A, CDH4-D134A) inhibited the radial migration of cortical neurons (Fig. 3D,E) providing evidence that cadherin-mediated adhesion is crucial for this process. Consistent with this finding, we observed by transmission electron microscopy the formation of adherens-like junctions ahead of the nucleus of migrating wild-type neurons (Fig. 3F,G).

### Regulation of radial migration by catenins and PTP1B

The cytoplasmic domains of CDH2 and CDH4 are identical in sequence and recruit large protein complexes comprising crucial mediators of cadherin function in adhesion and signaling, including α-catenin, β-catenin, vinculin and EPLIN (also known as LIMA1) (Hirano and Takeichi, 2012; Pokutta and Weis, 2007). We wanted to identify the downstream effectors that mediate cadherin function in migrating neurons. One difficulty in carrying out these experiments is the observation that CDH2 and catenins are essential for the formation of adherens junctions between RGCs. Perturbation of their function in RGCs leads to disruption of the ventricular neuroepithelium and gross morphological changes in the developing cortex (Gil-Sanz et al., 2014; Kadowaki et al., 2007; Lien, 2006). To circumvent this problem, we aimed at inactivating cadherin effectors specifically in migrating neurons.

**Fig. 4.**
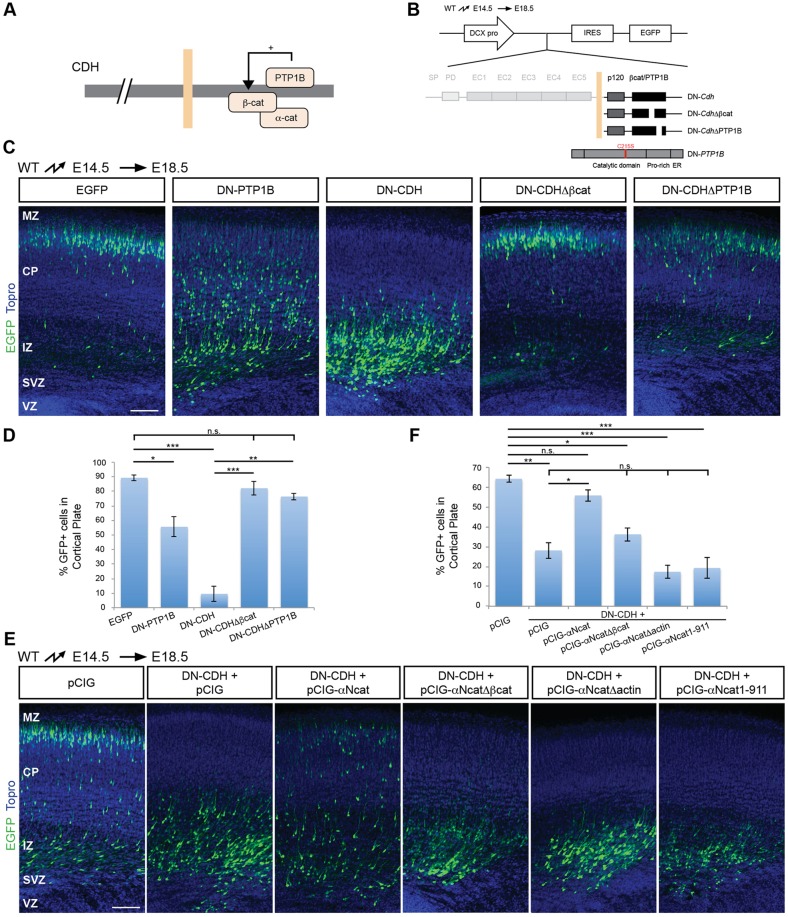
**The cadherin-catenin complex regulates migration.** (A) Diagram of the cadherin-catenin complex highlighting protein-protein interactions. PTP1B regulates interactions between cadherins and β-catenin. (B) Illustration of the strategy to test the relevance of CDH2-β-catenin interaction for neuronal migration. Different mutated forms of CDH2 and a dominant-negative (DN) PTP1B were expressed by *in utero* electroporation in migrating neurons at E14.5. The position of electroporated cells was analyzed at E18.5. DCX-pro, *Dcx* promoter fragment. ER, endoplasmic reticulum targeting domain. (C) Expression of DN-CDH, but not of DN-CDHΔβcat or DN-CDHΔPTP1B drastically affects migration. Expression of a DN-PTP1B with a mutation in the catalytic site also impairs migration. Electroporated neurons are shown in green, TO-PRO-stained nuclei in blue. (D) Quantification of the percentage of neurons that enter the CP. At least four animals from three separate experiments were analyzed for each condition. The data represent mean±s.e.m. n.s., not significant, *P<0.01, **P<1×10^−6^, ***P<1×10^−7^ by Bonferroni post-hoc analysis after one-way ANOVA. (E) Rescue experiments to address the importance of αN-catenin for neuronal migration. Full-length αN-catenin co-electroporated with DN-CDH rescued the migration defect caused by expression of DN-CDH alone. Mutated αN-catenin lacking binding sites for β-catenin (pCIG-αNcatΔβcat) or actin (pCIG-αNcatΔactin; pCIG-αNcat1-911) did not rescue the migration defect caused by expression of DN-CDH alone. Electroporated neurons are shown in green, TO-PRO-stained nuclei in blue. (F) Quantification (mean±s.e.m.) of the data in E. n.s., not significant, *P<0.001, **P<0.0001, ***P<1×10^−6^ by Bonferroni post-hoc analysis after one-way ANOVA. For each condition in D and F, neurons were counted in three brain slices from each of four animals obtained from three independent electroporation experiments. MZ, marginal zone. Scale bars: 100 μm.

Unfortunately, it was difficult to inactivate CDH2 and CDH4 expression completely in migrating neurons using DCX-Cre (Fig. S4). We also failed to achieve efficient protein knockdown of α/β-catenins by expressing shRNAs in wild-type neurons or by expressing Cre in neurons carrying floxed alleles, presumably because the half-life of the proteins was too long to achieve efficient protein depletion in the relatively short time-frame between terminal differentiation and onset of migration. We therefore took a different approach that allowed us to test whether interactions between different proteins and cadherins are crucial for radial migration. Phosphorylation of several Tyr residues in β-catenin inhibits interactions between β-catenin and CDH2 (Fig. 4A) (Balsamo et al., 1996; Qi et al., 2006). The protein phosphatase PTP1B binds to the cytoplasmic domain of classical cadherins and dephosphorylates β-catenin, thus facilitating cadherin binding to β-catenin (Balsamo et al., 1998). We therefore generated a dominant-negative (DN) form of PTP1B (C215S) that competes with wild-type PTP1B for binding to the cadherin cytoplasmic domain but no longer dephosphorylates β-catenin (Fig. 4B) (Balsamo et al., 1998). This construct was expressed in migrating neurons at E14.5 using the DCX-EGFP expression vector. Expression of the catalytically inactive PTP1B protein strongly impaired radial migration, with neurons remaining in the SVZ, IZ and lower part of the CP by E18.5 (Fig. 4C,D).

To confirm these findings, we took advantage of the DN-CDH construct (Fig. 4B), which consists of the conserved cytoplasmic domain of classical cadherins but lacks their extracellular domain (Franco et al., 2011). DN-CDH is thought to sequester the proteins that normally interact with the cytoplasmic domains of endogenous cadherins (Fujimori and Takeichi, 1993; Kintner, 1992; Ozawa and Kobayashi, 2014) and interferes with the delivery of endogenous cadherins to the plasma membrane (Nieman et al., 1999; Ozawa and Kobayashi, 2014). Consistent with earlier observations (Franco et al., 2011; Gil-Sanz et al., 2013; Kawauchi et al., 2010), neuron-specific expression of DN-CDH strongly inhibited radial neuronal migration (Fig. 4D,E). We hypothesized that mutations in DN-CDH that disrupt interactions with known binding partners such as β-catenin or PTP1B would mitigate this dominant-negative effect. We generated mutant constructs lacking the binding site for β-catenin (DN-CDHΔBCAT) or PTP1B (DN-CDHΔPTP1B) (Fig. 4B) (Xu et al., 2002). DN-CDHΔBCAT no longer sequesters β-catenin, whereas DN-CDHΔPTP1B no longer sequesters PTP1B from endogenous cadherins thus no longer preventing their dephosphorylation and interaction with β-catenin. Importantly, DN-CDHΔBCAT and DN-CDHΔPTP1B did not affect migration (Fig. 4C,D), suggesting that PTP1B-controlled interactions between CDH2/4 and β-catenin are crucial for radial neuronal migration.

### α-catenins and radial migration

Studies of cell migration in cultured cells have revealed the importance of the actin cytoskeleton in controlling cell motility (reviewed by Blanchoin et al., 2014). Radially migrating neurons in the neocortex express β-catenin and αN-catenin (CTNNA2) (Fig. 4A). At adhesion sites, β-catenin and αN-catenin form a complex that is linked via αN-catenin to the actin cytoskeleton (Hirano and Takeichi, 2012), thus providing a potential link between cadherins at the cell surface and the actin cytoskeleton within the leading processes of radially migrating neurons. To test the hypothesis that β-catenin-dependent recruitment of α-catenin is required for radial migration, we co-expressed DCX-DN-CDH and full-length αN-catenin in migrating neurons. Previous studies suggest that levels of β-catenin are relatively high in cells in which β-catenin contributes both to cell adhesion and to Wnt signaling (Brembeck et al., 2006; Bullions and Levine, 1998). DN-CDH therefore probably affects adhesion by recruitment of the β-catenin/αN-catenin complex, where αN-catenin is the rate-limiting component. We therefore reasoned that increased levels of αN-catenin would increase the total pool of the β-catenin/αN-catenin complex and thus alleviate the dominant-negative effect caused by recruitment of endogenous αN-catenin to DN-CDH. Consistent with this model, overexpression of αN-catenin significantly rescued the migratory defect caused by expression of DN-CDH alone (Fig. 4E,F). No rescue was observed when DN-CDH was co-expressed with αN-catenin carrying a mutation in a region that is crucial for interactions with β-catenin (Fig. 4E,F), or carrying mutations affecting either of the two domains that are required to mediate interactions with actin (Fig. 4E,F; Fig. S5). These findings suggest that cadherins regulate radial migration at least in part by β-catenin-dependent recruitment of αN-catenin and subsequent effects on the actin cytoskeleton.

### Defects in nucleokinesis

**Fig. 5.**
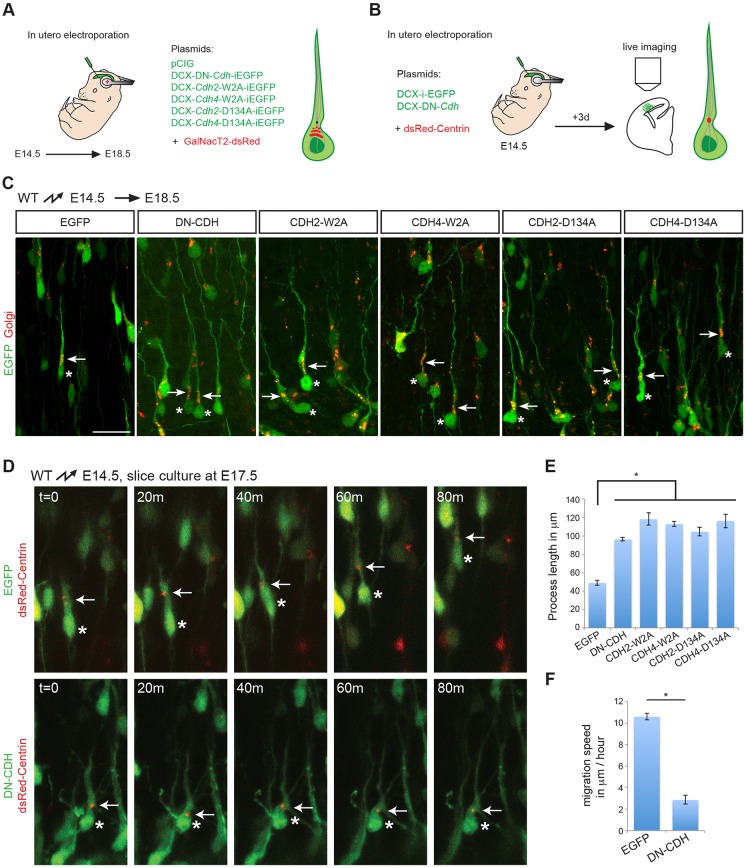
**Inhibition of nuclear and centrosomal movement.** (A) Diagram of experimental strategy to analyze neuronal morphology and nucleokinesis. DN-CDH and adhesion-deficient *Cdh2* and *Cdh4* constructs were co-electroporated with a fluorescence-tagged Golgi marker at E14.5. The morphology of electroporated neurons and the position of the Golgi apparatus were analyzed at E18.5, as shown in C. (B) Time-lapse imaging strategy to assess nuclear and centrosomal movement. Control or DN-CDH plasmids were co-electroporated with a fluorescence-tagged centrosomal marker at E14.5. Brains were processed for slice culture and live imaging at E17.5, as shown in D. (C) Neurons expressing DN-CDH or any of the adhesion-deficient cadherins extend long leading processes towards the CP (green). The Golgi apparatus (red) shows a polar localization in front of the nucleus in all experimental conditions. Nuclei are marked with asterisks, arrows point to the Golgi apparatus. Process length is quantified in E. Scale bar: 50 µm in C for C,D. (D) Time-lapse imaging of migrating neurons electroporated with a control plasmid (green, top panels) or DN-CDH (green, bottom panels). In control conditions, the centrosome (red) moves ahead of the nucleus into the swelling followed by nuclear movement. In DN-CDH-expressing cells, the centrosome stays close to the nucleus and neither organelle moves forward during the imaging period. The arrows indicate centrosomal position; nuclear position is marked by an asterisk. Data are representative of two independent experiments, each with two brains electroporated with control plasmid, two with DN-CDH and two with CDH2-D134A. Migration speed is quantified in F. (E) Quantification (mean±s.e.m.) of the process length from neurons expressing control plasmid, DN-CDH or adhesion-deficient cadherins, as in C. *P<0.001 by Bonferroni post-hoc analysis after one-way ANOVA. The number of processes measured for each condition was: 58 (EGFP, three brains), 40 (DN-CDH, three brains), 28 (CDH2-W2A, three brains), 42 (CDH4-W2A, four brains), 53 (CDH2-D134A, four brains) and 30 (CDH4-D134A, three brains). (F) Quantification of migration speed in neurons electroporated with control plasmid or DN-CDH. *P<1×10^−6^ by Student's *t*-test; 31 control cells (five brains) and 41 DN-CDH electroporated cells (six brains) from three different experiments were analyzed.

To define further the mechanism by which cadherins/catenins regulate radial migration, we visualized neuronal morphology and behavior at high resolution following perturbation of cadherin function in neurons (Fig. 5A). Cells that failed to migrate into the CP when cadherin function was perturbed by expression of DN-CDH, CDH2-W2A, CDH4-W2A, CDH2-D134A and CDH4-D134 did not show the multipolar morphology described in other cases when proteins involved in migration are mutated (reviewed by Ohtaka-Maruyama and Okado, 2015) (Fig. 5C). Instead, the neurons formed long leading processes that extended towards the MZ, but failed to translocate their nuclei along these processes (Fig. 5C,E). Co-expression of any mutant cadherin construct (DN-CDH2, CDH2W2A, CDH4W2A, CDH2D134A or CDH4D134A) with a Golgi marker (first 113 residues of GalNacT2 fused to DsRedex) (Fig. 5A) revealed localization of the Golgi apparatus in front of the nucleus, indicative of normal neuronal polarization (Fig. 5C). However, the leading processes of the polarized cells were abnormally long, thin and wavy (Fig. 5C,E), suggesting that they could extend towards the MZ but could not maintain tight contact with RGC fibers. Consistent with this finding, when we stained histological sections with nestin to reveal the glial scaffold, we observed that the leading processes of neurons expressing mutant cadherins were less well aligned with glial fibers compared with controls (Figs S6-S8).

Next, we analyzed cell behavior by time-lapse video microscopy. During glia-guided migration, neurons first extend a leading process, then a swelling appears in the process and the centrosome moves into it, followed by the nucleus (Schaar and McConnell, 2005; Solecki et al., 2004; Tanaka et al., 2004). To monitor centrosome behavior in real time, we expressed by *in utero* electroporation DN-CDH or control EGFP together with a centrosomal marker (DsRedex-Centrin) (Fig. 5B) in migrating neurons. Control neurons behaved as expected, with the centrosome moving into the leading process and the nucleus following (Fig. 5D, upper panels; Movie 1; Fig. 5F). By contrast, following perturbation of cadherin function, the centrosome remained close to the nucleus and the centrosome and nucleus failed to move forward into the leading process (Fig. 5D, lower panels; Movie 2; Fig. 5F). These findings suggest that in the absence of normal cadherin-mediated interactions with RGCs, nucleokinesis is perturbed.

### Defects in the leading processes of migrating neurons

**Fig. 6.**
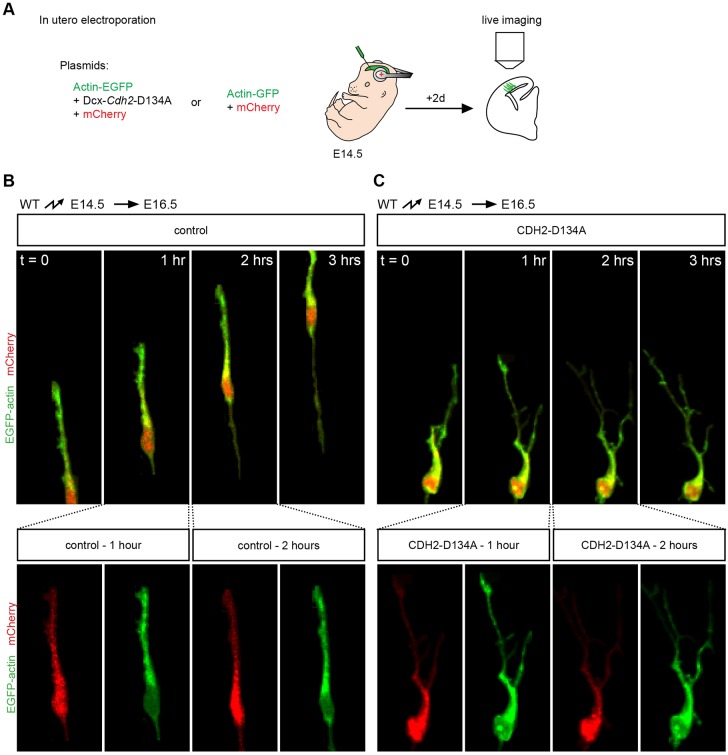
**Formation of lateral protrusions containing F-actin.** (A) Actin-GFP and mCherry were co-electroporated at E14.5 with or without DCX-*Cdh*2-D134A. Brains were processed for slice culture and live imaging at E16.5. (B) Actin (green) in control electroporated neurons is confined in a single leading process that shows no branching. The length of the leading process remains constant as the nucleus (red) moves forward. Bottom panels show individual channels for EGFP and m-Cherry for the 1- and 2-h images. (C) In neurons expressing CDH2-D134A, actin dynamics appear increased, with many transient actin-rich side branches (green) being created along the leading process. Note that the nucleus does not translocate and the neurons do not move forward, resulting in longer and thinner leading processes. Bottom panels show individual channels for EGFP and m-Cherry for the 1- and 2-h images. Experiments were performed five times, for a total of 17 control and 21 DN-CDH electroporated brains.

Proper organization of the actin cytoskeleton is crucial for centrosomal and nuclear movement during radial migration (Schaar and McConnell, 2005; Solecki et al., 2009; Tsai et al., 2007). We hypothesized that cadherin effects on nuclear migration were mediated at least in part by α/β-catenin-dependent effects on the assembly/stability of the actin cytoskeleton. We therefore analyzed the organization of the actin cytoskeleton in neurons that had been electroporated to express CDH2-D134A and an actin-EGFP fusion protein (Fig. 6A) (Murakoshi et al., 2008). These experiments were carried out in the first few days after electroporation, when the leading processes start to emerge from migrating neurons. In neurons expressing actin-EGFP and a control plasmid, F-actin was present abundantly in the leading process ahead of the nucleus (Fig. 6B; Movie 3). In neurons expressing CDH2-D134A, F-actin staining was detectable in addition in fine lateral branches that emanated from the leading process. These fine lateral branches were prominently visible in the neurons with perturbed cadherin function during the first few days following electroporation by labeling F-actin with actin-EGFP (Fig. 6C; Movie 4) but could not be resolved easily several days later with cytoplasmic EGFP in fixed tissue (Fig. 5C). Qualitatively similar results were observed when we expressed DN-CDH instead of CDH2-D134A in migrating neurons (data not shown). These findings suggest that normal cadherin-mediated adhesion is crucial for maintaining an organized actin cytoskeleton in leading processes and for restraining the formation of side branches rich in F-actin.

**Fig. 7.**
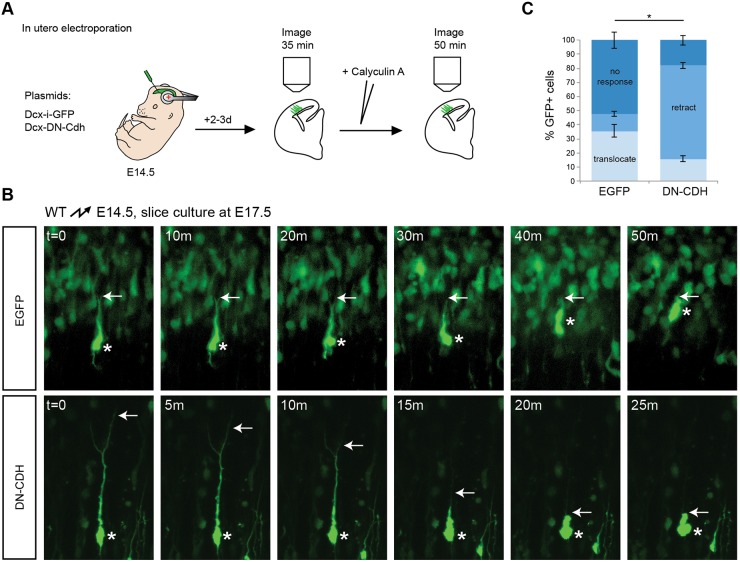
**Cadherin adhesion provides traction for radial migration.** (A) Illustration of the strategy to test whether cadherin is necessary for sustaining the traction forces that are required for nucleokinesis. Control or DN-CDH-expressing plasmids were electroporated at E14.5. Brains were processed 48-72 h later for slice culture and live imaging. Growth medium was replaced with medium containing 10 nM calyculin A about 30 min into the imaging session, and imaging was continued for 50 min. (B) Neurons electroporated with DCX-i-EGFP either do not respond to calyculin A, or tend to translocate their nucleus into the leading process (upper panels). The majority of neurons expressing DN-CDH retract their leading process in response to calyculin A (bottom panels). The arrows point to the tip of the leading process; nuclear position is indicated by an asterisk. This experiment was repeated five times. *t*=0 refers to the time when calyculin A was added. (C) Quantification of the response to calyculin A treatment. *P<0.01 for all three possible outcomes by Student's *t*-test (*P*<0.001 for no response; *P*<1×10^−7^ for retraction and *P*<0.01 for translocation). A total of 119 control and 195 DN-CDH electroporated neurons from three (control) and four (DN-CDH) brains were analyzed.

We reasoned that cadherin adhesion sites might provide the stable anchor points for F-actin that are necessary to sustain the contractile forces exerted on the cytoskeleton during nucleokinesis. When cadherin adhesion sites are weakened, the cytoskeleton may be unable to sustain the force necessary for the movement of the centrosome and nucleus. To test this model, we expressed DN-CDH in migrating neurons at E14.5. We prepared brain slices at E16.5 to monitor cell behavior by live imaging prior to and after the addition of calyculin A (Fig. 7A), an activator of actomyosin contractility that stimulates nuclear translocation in neurons (Solecki et al., 2009). As expected, calyculin A treatment of control EGFP-expressing cells stimulated nuclear translocation into the leading processes of radially migrating neurons (Fig. 7B, upper panels; Fig. 7C; Movie 5). By contrast, when calyculin A was added to neurons expressing DN-CDH, the vast majority of the leading processes rapidly collapsed (Fig. 7B, lower panels; Fig. 7C; Movie 6). These findings suggest that in the absence of stable cadherin-mediated adhesion sites, the cytoskeleton cannot sustain the forces that are exerted during nucleokinesis, thus leading to retraction of the leading processes.

### Functional interactions between cadherins and LIS1

**Fig. 8.**
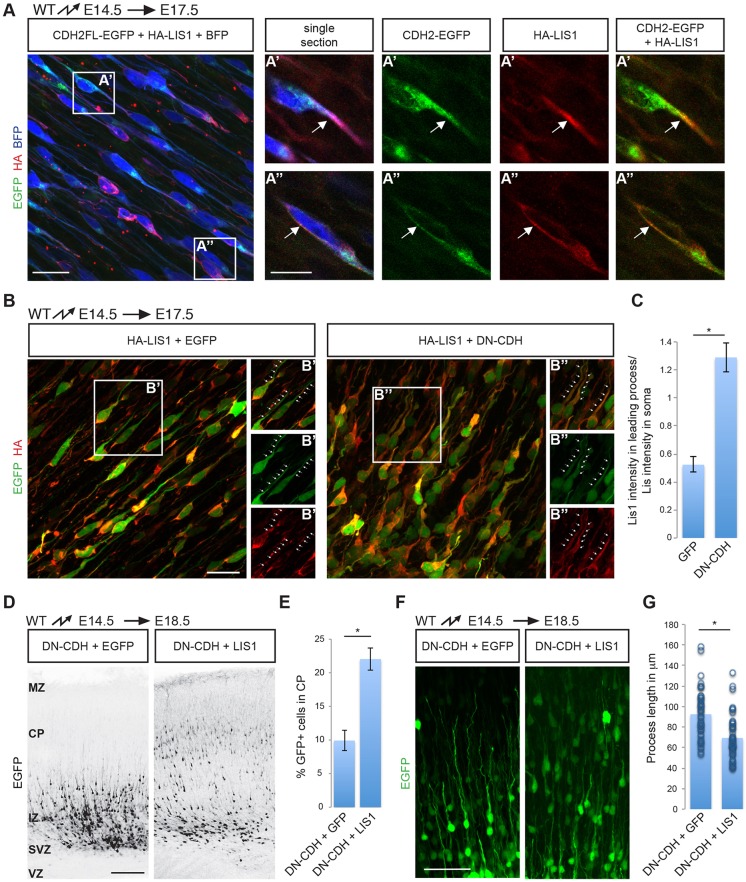
**Functional interactions between CDH2 and LIS1.** (A) Stack of confocal images of neurons expressing CDH2 (green), LIS1 (red) and BFP. EGFP-tagged CDH2 was co-electroporated with HA-tagged LIS1 and BFP at E14.5. CDH2 was visualized at E17.5 by EGFP fluorescence and LIS1 by staining with HA antibodies. Panels on the right are single confocal sections of the areas marked as A′ and A″. CDH2-EGFP and HA-LIS1 colocalize in the leading process (A′) and cell soma (A″). Arrows point to the leading processes of several neurons. (B) LIS1 localization is altered in neurons expressing DN-CDH. HA-tagged LIS1 was co-electroporated with either a control plasmid or DN-CDH at E14.5 and brains were analyzed 3 days later. DN-CDH-expressing neurons show increased HA-LIS1 staining in the leading process. B′ and B″ panels are single and combined channel images of the boxed areas in the main image. Dotted lines indicate leading processes. (C) Quantification of the relative HA (red) average fluorescence intensity in the leading processes versus soma of HA-LIS1 and control or DN-CDH co-electroporated neurons. *P<0.001 by Student's *t*-test. Fluorescence intensity was measured in 53 (control, four different brains) and 59 (DN-CDH, four different brains) neurons. (D) Co-expression of DCX-*Lis1* partially rescues the migration defect caused by expression of DN-CDH. DN-CDH was co-electroporated either with a control plasmid or with DCX-*Lis1*-i-EGFP at E14.5 and the position of the electroporated cells was assessed at E18.5. Electroporated neurons are shown in black. (E) Quantification (mean±s.e.m.) of the data in D. *P<0.01 by Student's *t*-test. Neurons were counted in three brain slices from each of four animals obtained from three independent electroporation experiments. (F) High magnifications of the neurons expressing DN-CDH+control plasmid or DN-CDH+LIS1. Note the difference in the length of the leading processes between the two conditions. (G) Quantification of process length of the neurons in F. *P<0.01 by Student's *t*-test. Process length was measured in 44 (DN-CDH+EGFP, four different brains) and 48 (DN-CDH+DCX-Lis1, five different brains) neurons. MZ, marginal zone. Scale bars: 20 μm (A,B); 10 μm (A′,A″); 100 μm (D); 50 μm (F).

Knockdown of LIS1 (also known as PAFAH1B1) leads to a similar defect in radial migration as reported here, in that the LIS1-deficient neurons form elongated leading processes but fail to translocate their nucleus along these processes (Tsai et al., 2005; Youn et al., 2009). LIS1 is thought to act in concert with its binding partner dynein 1 to regulate migration by effects on microtubules, which are targeted towards sites of cell-cell adhesion where dynein interacts with β-catenin (Ligon et al., 2001). Since proper organization of the microtubule cytoskeleton is crucial for nuclear migration, we hypothesized that defects in cadherin signaling might also affect microtubules, where the dynein-LIS1 complex might provide a link between microtubules and cadherins. To test this model, we first determined whether LIS1 and CDH2 colocalize in migrating neurons. Since no suitable antibodies for LIS1 were available for high-resolution immunolocalization studies, we generated expression vectors for a GFP-tagged version of CDH2 and a HA-tagged version of LIS1. We co-electroporated the constructs into migrating neurons at E14.5, together with BFP to delineate the neuronal cell bodies and processes (Fig. 8A). We then used EGFP fluorescence and antibodies to HA to analyze the distribution of CDH2-EGFP and HA-LIS1 at E17.5 during the active migration phase of the electroporated neurons. CDH2-GFP and HA-LIS1 colocalized in the cell bodies and the leading processes of radially migrating neurons (Fig. 8A). Next, we determined whether expression of DN-CDH did alter LIS1 localization in migrating neurons. DN-CDH-expressing cells showed significantly more LIS1 in their leading processes compared with control neurons (Fig. 8B,C). In addition, neurons overexpressing LIS1, although still able to migrate into the cortical plate, displayed altered leading processes that appeared thinner and shorter than those of control cells. However, neurons that had been electroporated with DN-CDH showed no such alterations in their leading process when they overexpressed LIS1 (Fig. S9). Strikingly, when we co-expressed DCX-LIS1 with DN-CDH, the migratory defect caused by expression of DN-CDH was significantly but not completely rescued (Fig. 8D,E). Our electroporation of DCX-LIS1 alone did not change the percentage of neurons reaching the CP after 4 days (Fig. S9), suggesting that CDH2 and LIS1 act in a common pathway to regulate radial migration. In fact, expression of DCX-LIS1 not only increases the number of DN-CDH electroporated neurons in the CP 4 days after electroporation, but it also decreases the length of their leading processes, bringing their length closer to that of control cells (Fig. 8F,G; compare with control cell length in Fig. 5E).

## DISCUSSION

Previous studies have shown that cadherins are required for the radial migration of neocortical projection neurons where they regulate multipolar migration, glial-guided motility and somal translocation (Franco et al., 2011; Gil-Sanz et al., 2013; Jossin and Cooper, 2011; Kawauchi et al., 2010). These findings have also provided insights into the signaling mechanisms by which cadherins regulate multipolar migration and somal translocation. However, the mechanisms by which cadherins regulate glial-guided motility have remained unclear. By perturbing cadherin signaling in radially migrating neurons at E14.5 and by monitoring neurons engaged in glial-guided motility, we demonstrate that CDH2 and CDH4 act via PTP1B as well as α- and β-catenin to regulate glial-guided motility. More specifically, our findings suggest that neurons with perturbed cadherin signaling polarize in the IZ and form long leading processes but they subsequently fail to translocate their nuclei and cell bodies forward. Our findings also suggest that actin-binding sites in α-catenin and crosstalk with LIS1 are crucial for cadherin function, thus suggesting that effects of cadherins are mediated at least in part by the cytoskeleton.

CDH2 and CDH4 are expressed in the developing cortex by RGCs and migrating neurons, and we show that both cadherins cooperate to regulate glial-guided motility, presumably by regulating interactions of neurons with RGCs. CDH2 and CDH4 interact homophilically and heterophilically, possibly generating adhesion complexes of distinct strength (Shan et al., 2000). When the adhesive function of either cadherin is weakened by point mutations in their extracellular domains, nucleokinesis is affected. Conversely, overexpression of wild-type CDH2 and CDH4, which probably increases adhesive strength, inhibits radial migration. Our findings suggest that precise control of adhesive strength is crucial for the formation of contacts between RGCs and migrating neurons that are sufficiently stable to withstand the tensile forces within the cytoskeleton during nucleokinesis, but that are also sufficiently dynamic to permit remodeling of cell-cell contacts during forward moving of the cell body.

Our findings also suggest that PTP1B, β-catenin and α-catenin are required to mediate cadherin functions during migration. PTP1B probably acts in radially migrating neurons in a similar way as it does in the formation of adherens junctions in epithelia, where it regulates interactions of β-catenin with cadherins by dephosphorylating β-catenin (Balsamo et al., 1998). Our findings also suggest that α-catenin mediates interactions of the cadherin/β-catenin complex with the actin cytoskeleton, because the actin-binding domains of α-catenin are required for its function in radial migration. Importantly, nucleokinesis of radially migrating neurons depends on the actin cytoskeleton, and actomyosin contractility increases ahead of the nucleus just before nucleokinesis (Solecki et al., 2009). Blebbistatin or jasplakinolide treatment, or myosin knockdown stop the forward movement of the nucleus, indicating that actomyosin contractility is crucial for nucleokinesis (Solecki et al., 2009; Tsai et al., 2007). Our findings are consistent with a model in which cadherins provide traction points for the leading processes of migrating neurons that permit transformation of contractile force into forward movement of the nucleus. Therefore, when actomyosin contractility is enhanced in neurons with weakened cadherin adhesion sites, their leading processes collapse. Further studies will be necessary to define the mechanism that mediates regulatory crosstalk between cadherins and the machinery that generates contractile force within the cytoskeleton.

Nucleokinesis is dependent not only on the actin cytoskeleton but also on microtubules, and interference with the function of the microtubule-binding proteins LIS1 or dynein halts centrosomal and nuclear movement (Tsai et al., 2007). LIS1, dynein, SUN1/2 and SYNE1/2 are thought to connect microtubules to the nuclear envelope (Tanaka et al., 2004; Zhang et al., 2009), and neurons electroporated with shRNAs targeting LIS1 fail to translocate their nucleus into the leading processes (Tsai et al., 2005). Interference with cadherin function causes a similar phenotype, suggesting that cadherins and LIS1 cooperate to regulate nucleokinesis. Strikingly, overexpression of LIS1 partially rescues the defect in radial migration caused by expression of DN-CDH. The mechanism that links cadherins and LIS1 needs further investigation. In epithelial cells, microtubules are targeted to adherens junctions through binding of dynein and β-catenin (Ligon and Holzbaur, 2007; Ligon et al., 2001), and LIS1 regulates dynein function, prolonging the time that individual dynein motors remain attached to microtubules (Huang et al., 2012). Prolonged attachment of microtubules to sites of cadherin-mediated adhesion could increase the kinesin-dependent delivery of cadherin molecules to the membrane (Meriane et al., 2002; Teng et al., 2005), strengthening those adhesions and thus promoting nucleokinesis.

Cadherins are crucial for the formation of stable adherens junctions between RGCs (Kadowaki et al., 2007) and for the formation of more dynamic contacts between RGCs and migrating neurons. The mechanisms that differentially regulate cadherin functions during different stages of neocortical development are unclear. Perhaps cadherins engage different signaling effectors for the formation of different types of adhesive contacts. RGCs in the VZ express αE-catenin (CTNNA1), whereas migrating neurons express αN-catenin (Ajioka and Nakajima, 2005; Stocker and Chenn, 2006). Interactions of α-catenins with actin involve other actin-binding proteins, such as EPLIN (Abe and Takeichi, 2008), α-actinin (Knudsen et al., 1995; Nieset et al., 1997) and vinculin (Weiss et al., 1998). Some of these effectors, including EPLIN, are strongly expressed in the cortical VZ but not in migrating neurons (not shown). EPLIN overexpression in migrating neurons impairs their radial migration (I.M.-G. and U.M., unpublished data). Thus, cadherins might engage different effectors to carry out their distinct functions during different stages of neocortical development. Interestingly, during somal translocation, the leading processes of migrating neurons extend large endfeet that form stable contacts with Cajal–Retzius cells. The formation of these contacts is regulated by reelin and involves cooperative interactions between CDH2 and members of the Ig superfamily, the nectins. Cadherins might also cooperate with distinct cell surface receptors during other stages of neocortical development, such as in the formation of adherens junctions or during glia-guided motility. Further studies will be necessary to investigate the extent to which cadherins act alone or in combination with other cell surface receptors to carry out their multiple functions in the developing neocortex.

## MATERIALS AND METHODS

### Mice

Experiments using mice [*Mus musculus* (Linnaeus, 1758)] were carried out under the oversight of an institutional review board. *Cdh2* floxed mice were purchased from The Jackson Laboratory (stock 007611). Mice carrying a floxed *Cdh4* gene were generated from knockout first embryonic stem cells (EPD0336_1_D06; Ryder et al., 2013), obtained from the European Conditional Mouse Mutagenesis Program (EUCOMM). Embryonic stem clones were injected into C57BL/6J blastocysts, and the resulting chimeras were mated to C57BL/6J females to obtain germ-line transmission. Genotyping of the offspring was carried out using the following primers: *Cdh4* forward primer, CGCTCAGCTTGGAAACAGCCCTGG; *Cdh4* reverse primer, GGTGGTGATCCTCTGCTCTCTGGG; and LAR3, CAACGGGTTCTTCTGTTAGTCC [sizes of PCR products: 439 bp (wild type); 287 bp (mutant)]. To remove the selection cassette of the knockout first mutation and generate the floxed allele, heterozygous F1 mice (*Cdh4^KO-flox/+^*) were mated with B6.Cg-Tg(ACTFLPe) mice (stock 005703; The Jackson Laboratory). Heterozygous offspring (*Cdh4^flox/+^*) were crossed to generate *Cdh4^flox/flox^* mice. Genotyping of the floxed animals was performed by PCR using: *Cdh4.1* forward primer, GAGAAACTACTCAAAGGTAGTGTGGG; *Cdh4.1* reverse primer, TGAACTGATGGCGAGCTCAGACC; size of PCR products: 300 bp (wild type); 266 bp (floxed). Mice on the C57BL/6J background were used as controls for *in utero* electroporations. For the staging of embryos and pups, midday of the day of the vaginal plug was considered as E0.5. Males and females were used for the experiments.

### Expression constructs

cDNAs were expressed in RGCs and neurons using the CAG-iEGFP vector containing the chicken β-actin promoter (CAG) and an IRES-EGFP (Hand et al., 2005). Neuron-specific expression was achieved using DCX-iEGFP, which contains the doublecortin promoter and an IRES-EGFP (Franco et al., 2011). DCX-Cre-i-GFP and DCX-mCherry have been described (Franco et al., 2011; Gil-Sanz et al., 2013). cDNAs for *Cdh*2, *Ctnna2*, *Ptp1b* and *Lis1* were amplified from embryonic mouse cDNA. In the blue fluorescent protein (BFP) expression vector, BFP expression was driven by the CAG promoter. For construct details, see Table S1.

### *In utero* electroporation and time-lapse imaging

Electroporations and time-lapse imaging were performed as described (Franco et al., 2011). Imaging was carried out using a Nikon C2 or a Zeiss LSM780 laser-scanning confocal microscope. For quantification, the percentage of EGFP^+^ cells located in different positions within the cortical wall was determined (mean±s.e.m.). At least four animals from three separate experiments were analyzed for each condition.

### *In situ* hybridization and transmission electron microscopy (TEM)

*In situ* hybridizations and TEM were carried out as described (Franco et al., 2012; Tiveron et al., 1996). *In situ* probes are summarized in Table S1. Bright-field images were captured using an Olympus AX70 microscope. Sections for TEM were examined on a Philips CM100 electron microscope (FEI) at 80 kV. Images were collected using a Megaview III CCD camera (Olympus Soft Imaging Solutions).

### Histology and immunostaining

Embryonic brains were dissected and fixed in 4% paraformaldehyde overnight at 4°C. Brains were sectioned coronally at 100 µm with a vibrating microtome (VT1200S; Leica). Immunostaining was performed as described (Belvindrah et al., 2007; Franco et al., 2011, 2012; Gil-Sanz et al., 2013). For antibodies, see Table S2. Nuclei were stained with DAPI or TO-PRO (Thermo Fisher Scientific). Sections were mounted on slides with Prolong Gold mounting medium (Thermo Fisher Scientific). Images were captured using a Nikon C2 or a Zeiss LSM780 confocal microscope. Cellular measurements were performed using ImageJ software.

### Calyculin A treatment

C57Bl6/J mice were electroporated with DCX-iGFP or DCX-DN-CDH-iGFP at E14.5. Slices (200 µm) were cut at E16.5-E17 and imaged one frame every 5-6 min through a *z*-stack of 10 µm with a 2.5 µm step (40× long working-distance objective on a Nikon A1 laser-scanning confocal microscope). After five to eight frames without calyculin A, a 10 µl drop of 100 nM calyculin A (Sigma-Aldrich) was added directly to the top of each slice and imaging was continued with the same settings.

### Methodology and statistics

Sample size (*n*) was determined using online software (https://www.ai-therapy.com/psychology-statistics/), providing parameters for the type of analysis being performed. For a Cohen's d value of 3.3, obtained if there is a 1.5-fold change between two groups with a standard deviation of 15%, a sample size of *n*=4 provides a power of 0.9. All electroporations included control plasmids, and controls were designed considering potential plasmid dilution in the experimental condition. Exclusion criteria for electroporated brains included samples with abnormal morphology due to experimental manipulation, and litters in which control electroporations clearly deviated from the expected neuronal distribution pattern. The data presented meet normality criteria and were analyzed using either one-way ANOVA followed by post-hoc Bonferroni tests (multiple samples) or Student's *t*-test (two samples).
